# Exploring Muscle Activation during Nordic Walking: A Comparison between Conventional and Uphill Walking

**DOI:** 10.1371/journal.pone.0138906

**Published:** 2015-09-29

**Authors:** Barbara Pellegrini, Leonardo Alexandre Peyré-Tartaruga, Chiara Zoppirolli, Lorenzo Bortolan, Elisabetta Bacchi, Hélène Figard-Fabre, Federico Schena

**Affiliations:** 1 CeRiSM (Research Centre of Mountain Sport and Health), University of Verona, Rovereto, Italy; 2 Department of Neurological and Movement Sciences, University of Verona, Verona, Italy; 3 School of Physical Education, Universidade Federal do Rio Grande do Sul, Porto Alegre, Brasil; 4 Department of Medicine, University of Verona, Verona, Italy; 5 Departmental Office of Sports, General Council of High Pyrenees, Tarbes, France; University of Utah, UNITED STATES

## Abstract

Nordic Walking (NW) owes much of its popularity to the benefits of greater energy expenditure and upper body engagement than found in conventional walking (W). Muscle activation during NW is still understudied, however. The aim of the present study was to assess differences in muscle activation and physiological responses between NW and W in level and uphill walking conditions. Nine expert Nordic Walkers (mean age 36.8±11.9 years; BMI 24.2±1.8 kg/m^2^) performed 5-minute treadmill trials of W and NW at 4 km/h on inclines of 0% and 15%. The electromyographic activity of seven upper body and five leg muscles and oxygen consumption (VO_2_) were recorded and pole force during NW was measured. VO_2_ during NW was 22.3% higher at 0% and only 6.9% higher at 15% than during W, while upper body muscle activation was 2- to 15-fold higher under both conditions. Lower body muscle activation was similarly increased during NW and W in the uphill condition, whereas the increase in *erector spinae* muscle activity was lower during NW than W. The lack of a significant increase in pole force during uphill walking may explain the lower extra energy expenditure of NW, indicating less upper body muscle activation to lift the body against gravity. NW seemed to reduce lower back muscle contraction in the uphill condition, suggesting that walking with poles may reduce effort to control trunk oscillations and could contribute to work production during NW. Although the difference in extra energy expenditure between NW and W was smaller in the uphill walking condition, the increased upper body muscle involvement during exercising with NW may confer additional benefit compared to conventional walking also on uphill terrains. Furthermore, people with low back pain may gain benefit from pole use when walking uphill.

## Introduction

Nordic Walking (NW) is a form of physical activity where conventional walking is supported by the use of specially designed poles. According to the International Nordic Walking Federation (INWA), the correct technique in use of the poles involves a backward pole position during the loading phase, active and dynamic use of the poles, and control of the poles with the grip and strap. Use of the poles actively engages the upper body to propel the body forward during walking.

Oxygen uptake (VO_2_), heart rate (HR), [1,2,3,4,5,6] and blood lactate concentration [4] are all reportedly higher when walking with poles than without them. Energy expenditure at a given speed is about 20% higher when walking with poles [1,2,4], with the amount of the differences depending on technical and equipment-related factors. Energy expenditure is largely influenced by the technical execution of NW. Greater differences in energy expenditure between conventional walking and NW were found when the subjects adopted a vigorous NW technique [6] or refined their technique [3]. Energy expenditure also depends on pole weight [7] and length [6]. Moreover, the extra energy cost of using poles is related to ground surface conditions [8] and terrain slope, which explains the significant slope x pole use interaction effect on energy expenditure. A smaller difference in energy expenditure between walking with and without poles was found during walking on moderate (5%) [3] and steeper (~21%) inclines [6]. To our knowledge, no studies to date have explained the reason for this observation.

We can hypothesise that the increased energy expenditure during NW is due neither to the augmented arm swing nor to the increased hand-held weight. The first hypothesis is supported by a study reporting that metabolic energy expenditure was significantly higher (5–8%) during walking without arm swing than during normal walking [9]. The second is suggested by studies demonstrating that the use of heavier poles of up to 1.5 kg during NW [7] and hand-held weights in walking and running [10] have no effect on energy expenditure.

The higher energy expenditure when walking with poles can be attributed to greater activation of the upper body muscles engaged in poling action [7,11]. However, few studies have compared electromyographic (EMG) activity in NW and conventional walking. Less activation of the *vastus lateralis* and *gastrocnemius* muscles during NW compared to conventional walking was found across a wide range of walking speeds, while less activation of the *biceps femoris* was noted during NW at speeds faster than 6 km/h [5]. The study investigated only the *triceps brachii* muscle, and its activity was reported to increase significantly during NW. A more recent study comparing the effects of waking with and without poles in only one experimental condition [12] found remarkably increased activation of the *biceps brachii*, *triceps brachii*, *deltoid medius* and *latissimus dorsi* muscles but no significant differences in leg muscle activation during NW. In addition, although NW promoters claim that NW involves a substantial use of the trunk muscles, their activation during NW has not yet been studied.

Studies on upper body muscle activity in locomotion with pole propulsion for double poling in cross-country skiing [13,14,15] have reported high-to-medium muscle activation during flexion-extension of the hip, trunk, shoulder, and elbow in the poling phase during high-intensity double poling [14] and increased muscular activation in response to an increase in speed [13]. No direct comparison can be made with NW, however, because of the different poling times and patterns of joint motion in cross-country skiing locomotion, where trunk flexion is much greater and elbow extension during poling is preceded by rapid elbow flexion [14].

Uphill walking is known to elicit increased EMG activity in leg extensor muscles [16,17], and an interaction between incline and speed during walking has been demonstrated [18]; studies identifying the changes in muscle activation with grade for NW are lacking, however. The relative changes due to the degree of incline in the activation of lower and upper body muscles between walking with and without poles have not yet been investigated; therefore, no information supporting the practice of NW on uphill terrain is available. The main aim of the present study was to compare muscle activation during NW-based exercise and conventional walking in level and uphill conditions. Moreover, we wanted to determine whether the changes in muscle activation could explain the differences in oxygen consumption between NW and conventional walking and the changes with degree of incline. Our hypothesis was that NW elicits muscle activation patterns different from those of conventional walking, with greater activation of the muscles involved in poling action. We also hypothesised that, based on subjective reports by NW practitioners, the difference in trunk muscle activation between NW and conventional walking is small but still relevant. Finally, we hypothesised that, as noted in previous studies reporting a significant energy expenditure x slope interaction for pole walking [3,6], the differences in physiological response between NW and W in level and uphill walking conditions would be smaller in the uphill walking condition.

## Materials and Methods

### Subjects

The study population was 9 male NW instructors (mean age 36.8±11.9 years, height 1.78±0.10 m, body weight 75.8±5.8 kg, body–mass index [BMI weight in kg divided by height in meters squared] 24.2±1.8 kg/m^2^) licensed by the ANWI (Associazione Nordic Walking Italia) and with at least 2 years of experience in NW (mean 2.89±1.00 years). The general health status was normal; none had any health condition that could affect exercise capacity.

### Ethics Statement

The study was approved by the Ethical Committee of Verona University. All participants were informed verbally and in written form about the nature and procedures of the study before they gave their written consent to participate. They were also informed about their right to withdraw from the study at any time without the need to provide explanation.

### Experimental Procedure and Protocols

Tests were performed on a motorized treadmill with a belt surface 2.5 m wide and 3.5 m long (RL3500E, Rodby, Sweden). Subjects used NW poles (Exel, Nordic Walker, Espoo, Finland) equipped with special carbide tips to ensure appropriate grip with the treadmill belt surface. As recommended by the INWA, correct pole length was determined by multiplying the subject’s height in cm by 0.68 rounded down to the nearest 5 cm within a tolerance of 2.5 cm. The subjects performed 5-min tests on the treadmill under the following combination of conditions: conventional walking and NW on level (0%) and uphill walking at a 15% incline. These inclines were chosen because, according to the Nordic walking instructors, both are common conditions encountered during NW. Walking speed was maintained at 4 km/h in each slope and gait condition because it is the usual pace that can be comfortably sustained on either incline. During NW, all subjects used the diagonal technique, which is the most common NW technique worldwide and is characterized by contralateral leg and arm coordination. All tests were presented in random order.

### Measurements

Gas exchange and ventilatory parameters were collected breath-by-breath by means of a portable metabolic system (Cosmed K4b2, Rome, Italy) with the main sample unit attached to the chest and the battery pack on the back (800 g). The subjects wore a facemask (70 mL dead space) that directed the respiratory gases via the sampling tube to the analyzer. A low-resistance bidirectional turbine incorporated in the mask measured the breath volume. Before each test, the turbine was calibrated with a 3-L volume syringe (Cosmed, Rome, Italy); the gas analyzer was calibrated with ambient air (20.93% O_2_ and 0.03% CO_2_) and a known concentration of gasses (16.00±0.04% O_2_ and 5.00±0.01% CO_2_) (Air Liquide Italia S.p.A., Milan, Italy).

A portable EMG system (Myomonitor, Delsys Inc., Boston, MA, USA) recorded the surface EMG activity from seven upper body (*lumbar erector spinae* [ES], *trapezius* [TR], *latissimus dorsi* [LD], *deltoideus anterior* [DA], *biceps brachii* [BB], *caput lateralis* of *triceps brachii* [TB], and *rectus abdominis* [RA]) and five lower limb (*gluteus medius* [GM], *vastus lateralis* [VL], *biceps femoris* [BF], *gastrocnemius lateralis* [GL] and *tibialis anterior* [TA]) muscles of the arm dominant side of the body. Handedness was determined on the basis of self-reported hand use. We chose the lateral head of triceps because we wanted to isolate elbow extensor action. Parallel-bar EMG electrodes (DE-2.3, 5-mm single differential surface EMG sensor with two 1-mm Ag contacts 10 mm apart) were positioned longitudinally on the belly of each muscle with respect to the underlying muscle fibres in accordance with standard recommendations [19,20] to minimize cross-talk and geometrical artifacts [21]. For the *erector spinae* muscle, the electrode was attached at the height of the third lumbar vertebra on the dominant side, 2 cm lateral to the spinous processes [20]. A reference electrode was placed on the posterior aspect of the proximal epiphysis of the right radius. To minimize impedance, the skin was shaved, slightly abraded, degreased, and disinfected with alcohol before attaching the electrodes. The electrode wires were kept close to the skin with elastic nets to avoid artifacts due to movement.

The EMG signals were sampled at a frequency of 1000 Hz, hardware amplified (gain 1000 V/V ± 1%), band-pass filtered (20–450 Hz; 20 dB/oct) to remove noise, converted A/D and transmitted wirelessly (D-link WUA-1340 Wireless G USB adapter) to a computer for real-time data display and storage (EMGworks Acquisition Software, Delsys Inc.).

A foot switch (DC-F01, Delsys Inc.) was used to detect foot contact with the ground. The switch consists of a circular pressure sensitive resistive membrane (radius 1.27 cm) that was attached with double-layer tape to the skin on the rear part of the foot plantar surface under the heel. The signal was acquired with the same device used to sample and record the EMG signals.

Pole force was measured during NW with a lightweight single-axial load cell (Deltatech, Sogliano al Rubicone, Italy) mounted inside the poles under the handgrip. Analog signals from the force transducer were sampled at 100 Hz by means of a data acquisition board (NI DAQ-PAD-6016, 16 bit; National Instruments, Austin, TX, USA). Force transducers were dynamically calibrated a few minutes before each test using a load cell as a reference (546QD; DSEurope, Milan, Italy), as described previously [22]. Data collection for EMG, foot switches, and pole force was triggered by a digital signal in order to ensure synchronization between the force signals, the EMG signal, and the foot switch.

### Data Processing

The average value of oxygen consumption (VO_2_) was calculated over the last 30 s of each condition. The root mean square amplitudes (RMS) of the EMG signal were calculated for each muscle and for each complete gait cycle occurring within the 30-s acquisition period. Gait cycle was defined as the interval between two consecutive heel strikes of the foot on the investigated body side. Instants of heel strike were recognized when the signal of the foot sensor rose and passed a threshold value of 10% of the average signal measured during the acquisition period. For visualization purposes, the EMG signals were processed by an RMS moving window (window size 250 ms; stepwise value for value); each walking cycle was then standardized in time to 100 of the whole cycle. For each subject and muscle, the RMS values were normalized using the mean dynamic method [23] and expressed as percentage of the mean RMS calculated from the EMG signal obtained during conventional walking on level incline. Ensemble averages for each subject were calculated from 20 consecutive cycles and then used to calculate the ensemble average for the group. The magnitude of differences in RMS values between the gaits at a given incline were quantified and are reported as the percentage differences between NW and conventional walking expressed with respect to the conventional walking values. Similarly, the magnitude of the differences in RMS between the inclines for a given gait are reported as the percentage differences between the values obtained at inclines of 15% and 0% expressed with respect to the values at 0% incline.

For NW, the poling cycle was defined as beginning at pole ground contact and ending at the subsequent ground contact of the same pole. Pole contact and pole take-off were identified in the force data as the first point above and the first point below a force threshold of 4 N, respectively. The cycle time for poling action was calculated as the time between two subsequent pole contacts and the duration of the pole thrust action; poling time (PT) was calculated as the time between pole ground contact and pole take-off. The average poling force (PF) over the entire cycle was calculated by dividing the integral of the force–time curves by the duration of poling cycle time. Data were processed using Matlab 7.0 (MathWorks Inc., Natick, MA, USA) and Excel 2003 (Microsoft Corporation, Redmond, WA, USA).

### Statistical Analysis

The Shapiro–Wilk test for normality showed that the data were normally distributed. For each condition, the values for each subject are expressed as the mean of all the cycles during the 30-s period of data acquisition. Two-way repeated measures multivariate analysis (MANOVA) was applied to evaluate the influence of gait and incline on the parameters of interest. Assumption of sphericity was checked using Mauchly's test. A Holm–Bonferroni test to correct for alpha inflation was applied post hoc to confirm differences between the gaits at each degree of incline and between the degrees of incline at each gait. Statistical analyses were carried out using SPSS 15.0 Software for Windows (IBM SPSS Inc., Chicago, IL, USA). Statistical significance was set at p < .05.

## Results

**Fig 1:**
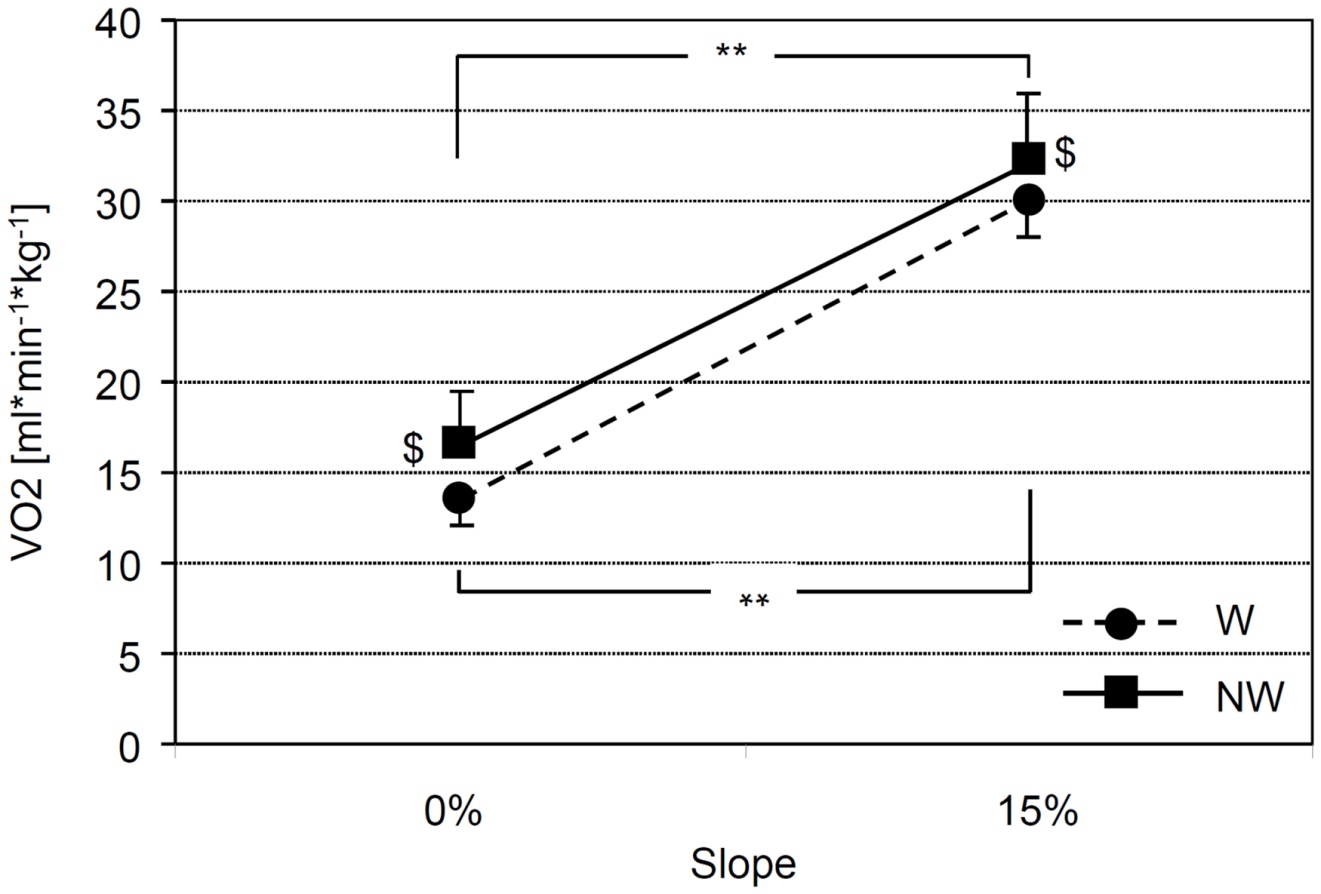
Oxygen uptake during conventional walking (W) and Nordic Walking (NW). VO_2_ values (mean ± standard deviation) as a function of slope for W (circles and solid line) and NW (squares and dashed line). $ indicates significant differences between gait at each slope (p < .05); ** indicates significant differences between inclines at 0% and 15% for each gait (p < .005).

Oxygen uptake (VO_2_) was significantly dependent on gait (NW vs. conventional walking [W]) (F(1,8) = 17.58; p = .003) and on the degree of incline (F(1,8) = 739.8; p < .001); a significant gait x uphill slope interaction was found (F(1,8) = 8.95; p = .017) (Fig 1). VO_2_ was significantly increased in the uphill walking condition during both NW (p < .001) and W (p < .001). In the level walking condition, VO_2_ was 22.6% greater during NW than W (p = .0013) and was 6.9% greater during NW than W in the uphill walking condition (p = .033) (Fig 1). Exercise intensity (expressed in METs) was 3.85 ± 0.42 for W and 4.72 ± 0.86 for NW in the level condition, corresponding to a moderate-intensity activity, and 9.18 ± 1.08 for W and 8.56 ± 0.58 for NW in the uphill condition, identifying a vigorous intensity activity for both gait modes [24].

**Fig 2:**
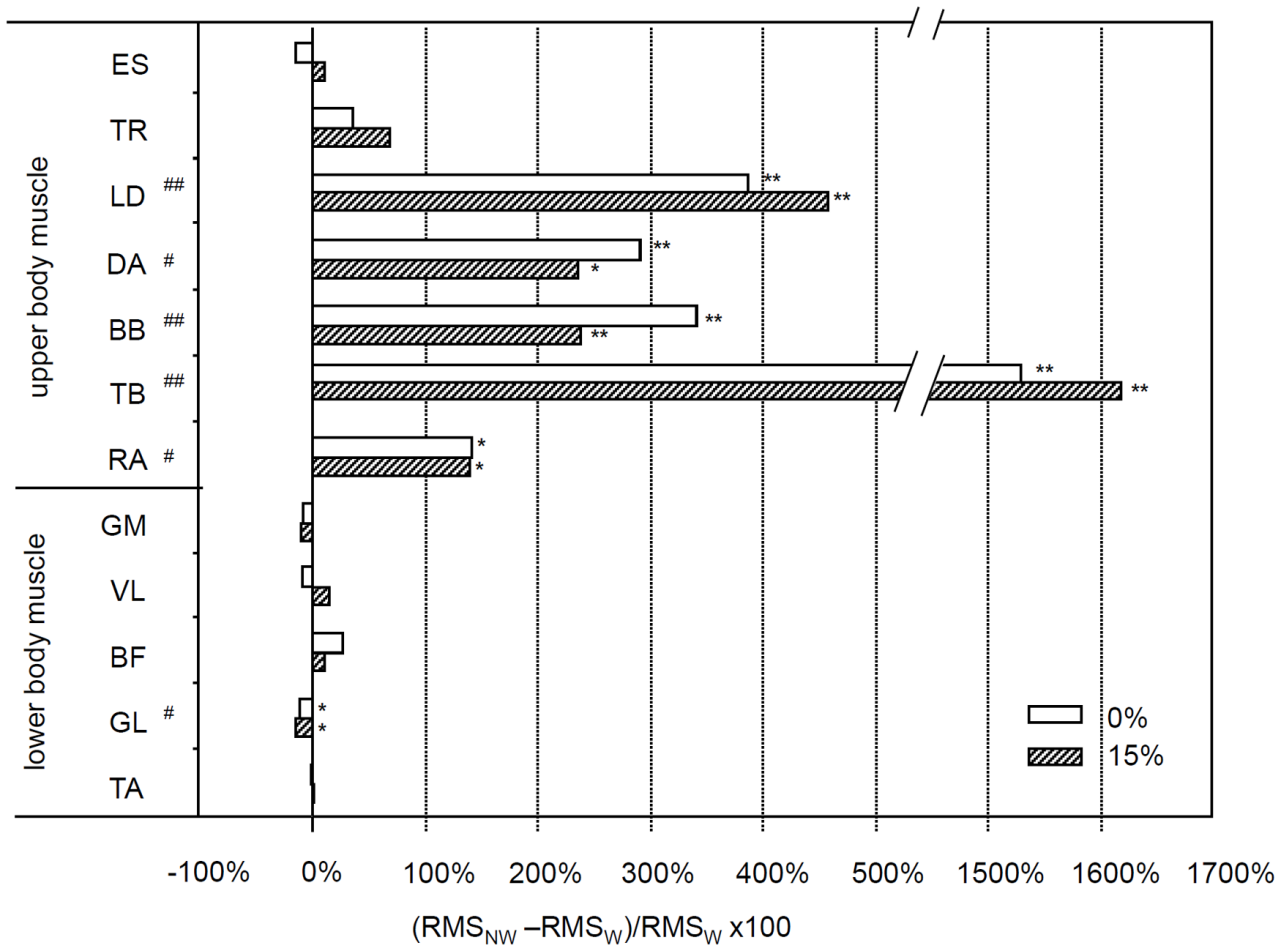
Percentage differences in EMG amplitude between NW and W. Percentage differences in RMS values of the EMG signal calculated as the differences between W and NW with respect to W for all muscles at 0% incline (empty bars) and 15% incline (gray filled bars). # p < .05 and ## p < .005 indicate a significant effect of gait on RMS values, as ascertained by gait x slope MANOVA; * p < .05 and ** p < .005 indicate significant differences between W and NW at each slope when the gait effect was significant.

A significant effect of gait on the RMS values for five of the seven upper body muscles was noted: LD (F(1,8) = 25.96; p < .001); DA (F(1,8) = 13.69; p = .006); BB (F(1,8) = 26.81; p < .001); TB (F(1,8) = 22.79; p < .001); and RA (F(1,8) = 10.51; p = .012) and for one of the lower body muscles, GL (F(1,8) = 11.95; p = .009). Post hoc analysis revealed that upper body muscle activation was increased from W to NW in both the level and uphill walking conditions; activation of the GL muscle was decreased in the level and uphill walking conditions, with a decrease in RMS values of 12% and 15%, respectively (Fig 2).

**Fig 3:**
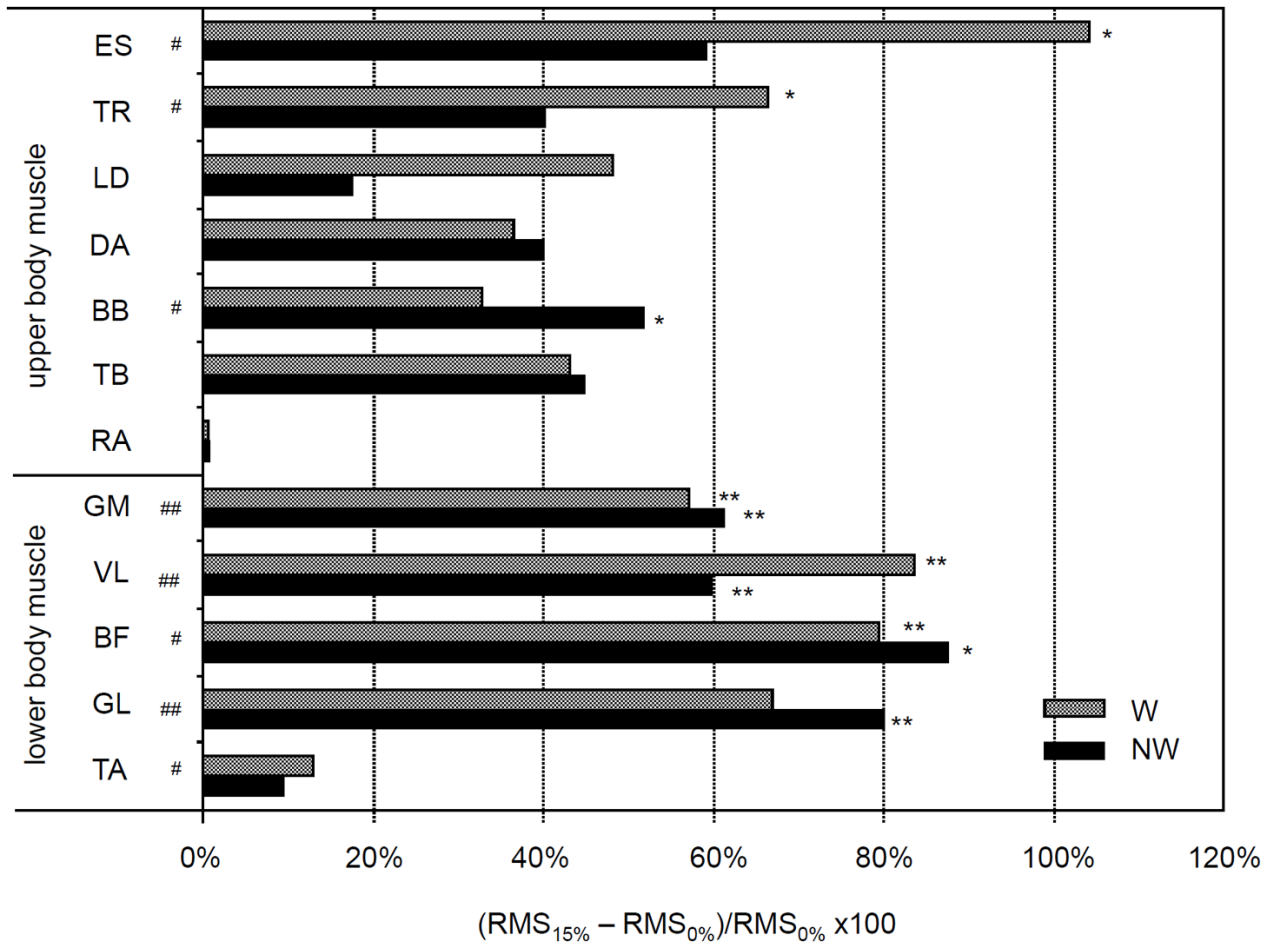
Percentage differences in EMG amplitude between uphill and level walking. Percentage differences in RMS values of the EMG signal calculated as the differences between the values measured at inclines of 15% and 0% with respect to 15% incline for all muscles during W (gray filled bars) and NW (dark filled bars). # p < .05 and ## p < .005 indicate a significant effect of the degree of incline on RMS values, as ascertained by gait x slope MANOVA. * p < .05 and ** p < .005 indicate significant differences between inclines at 0% and 15% for each gait when the gait effect was significant.

Fig 3 reports the inter-individual ensemble average of EMG values during W and NW in the level walking condition for those muscles where a significant gait effect was found. The increase in the EMG values of the LD and TB muscles was greater during NW than W mainly during the second half of the gait cycle, which corresponds to poling action of the arm. The EMG values for the BB and DA muscles were higher in both the poling and the recovery phase during NW, with a large standard deviation noted for the BB muscle in the first half of the cycle, indicating high between-subject variability during pole recovery. The EMG values for the RA muscle were increased throughout most of the cycle during NW. The EMG values for the GL muscle were decreased during NW in the final part of the stance phase during leg propulsion.

**Fig 4:**
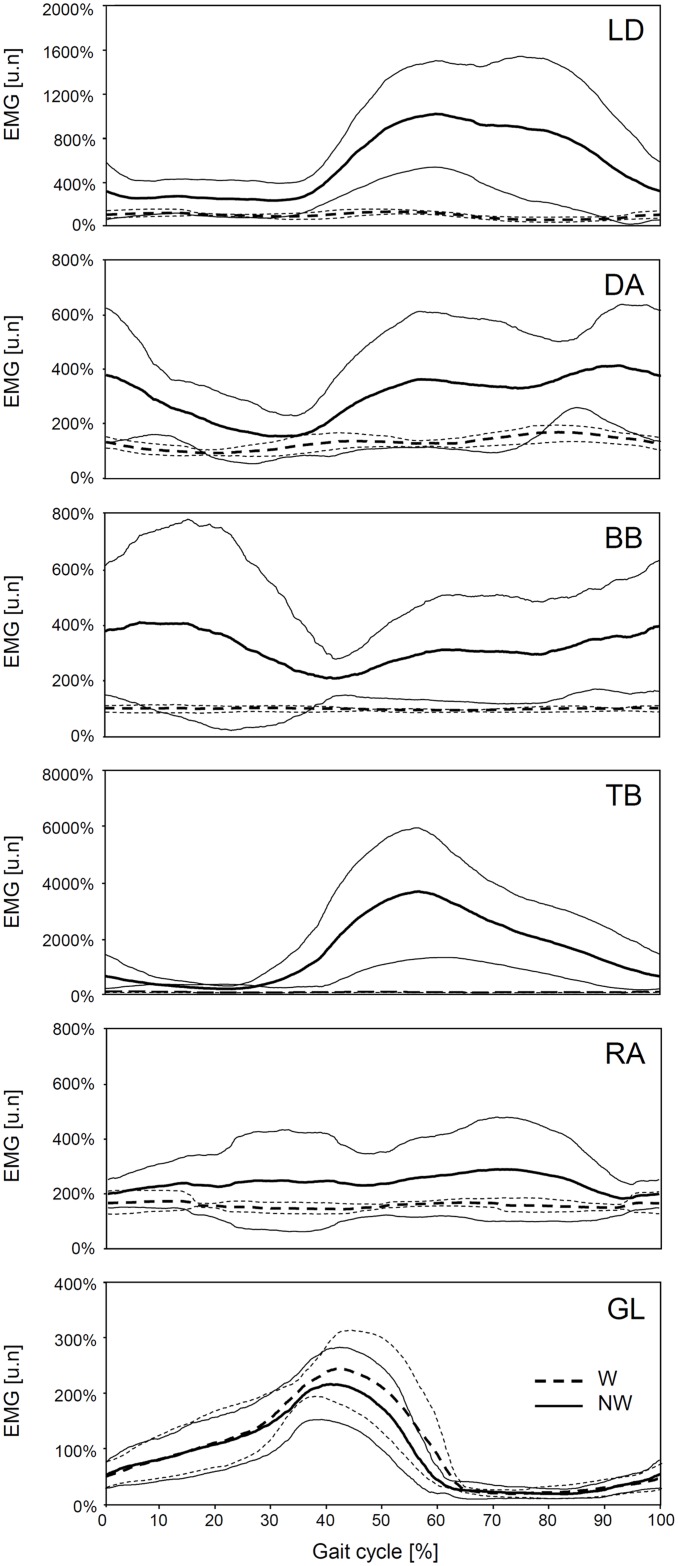
EMG curves for a gait cycle in conventional walking and Nordic Walking. Inter-individual ensemble average (±SD) of the RMS value of EMG signals during W (dashed line) and NW (solid line) in the level walking condition, normalized with respect to the mean values obtained from the main muscles during W. From upper to lower panel: LD latissimus dorsii, DA deltoideus anterior, BB biceps brachii, TB triceps brachii, RA rectus abdominis, GL gastrocnemius lateralis.

A significant effect of slope on the RMS values for three upper body muscles was noted: ES (F(1,8) = 5.84; p = .042); TR (F(1,8) = 13.71; p = .006); BB (F(1,8) = 8.49, p = 0.019), and for five lower body muscles: GM (F(1,8) = 43.1, p< .001); VA (F(1,8) = 13.40, p = .006); BF (F(1,8) = 9.87; p = .014); GL (F(1,8) = 22.33; p = .001); TA (F(1,8) = 6.39; p = .035). Post hoc analysis revealed that in all muscles for which the slope effect was significant there was a significant increase in the RMS values between level and uphill walking during both W and NW (Fig 4).

A significant gait x slope interaction in RMS values for the ES muscle was noted (F(1,8) = 22.93; p < .001); the increase in muscular activity was significant during W (104%; p = .0466) but not during NW (59%; p = .0879) (Fig 4). There were no significant differences in the average poling force values between the level and uphill walking conditions (14.24±7.40 N at 0% incline vs. 18.47±9.28 N at 15% incline; p = .096).

## Discussion

Nordic walking owes much of its growing popularity to the fact that it is advertised as physical activity that, as compared to plain walking, increases energetic expenditure by involving the upper body muscles. Our data confirm that oxygen consumption is higher during NW than conventional walking and show that walking with poles significantly increases activation of the upper body muscles involved in the poling action. Furthermore, the smaller difference in energy expenditure between conventional walking and NW in the uphill walking condition than during level walking was most likely due to less use of the poles to lift the body against gravity during propulsion. Uphill walking with poles may, however, reduce back muscle activation.

### Metabolic and EMG Differences in Nordic Walking vs Conventional Walking

Oxygen uptake was greater during NW than conventional walking at the same speed, with an estimated extra energy cost of about 23% during level walking at 4 km/h and of 7% during uphill walking. The metabolic data are in line with previous studies investigating energy expenditure [1,2,4] during NW on flat terrain. An extra energy cost of 68% was found in subjects using a vigorous NW technique [6], but no differences were noted when the subjects walked with hiking poles [25] or walked at a brisk speed (2 m/s) [7]. Therefore, the amount of increase in energy expenditure when walking with poles is not a universal value and it is more likely explained by the technique the subjects adopted and by proper pole use.

EMG muscle activity of the upper body muscles during NW was highest in the *triceps brachii* muscle, with an average 16-fold increase in muscle activation during NW as compared to conventional walking, and a 40-fold increase at the peak of activation during poling action, followed by the *latissimus dorsii* muscle, with an average 4-fold increase during NW as compared to conventional walking. Both the *triceps* and the *latissimus dorsii* muscles are involved in the poling phase during elbow extension and shoulder back extension, respectively. Because muscle activation is increased in the second part of the gait cycle, corresponding to the poling phase, it can be hypothesized that the increased activity of these two muscles concurs during poling force exertion.

A significant increase in *biceps brachii* and *deltoideus anterior* muscle activation during NW was also seen, with an average 2- to 3-fold increase throughout the entire gait cycle. As the *biceps brachii* serves to flex the elbow and the *deltoideus anterior* to medially rotate and stabilize the shoulder, we can hypothesize that they are not only involved in pole recovery during the arm swing phase but also work in coactivation with the *triceps brachii* and *latissimus dorsii* muscles during the poling phase so as to stabilise the elbow and shoulder joints. The increased activity of the *rectus abdominis* muscles throughout the entire gait cycle during NW may be imputable to a greater need for trunk stabilization. The higher oxygen consumption measured during NW as compared to conventional walking can be attributed to increased muscle activation primarily for thrust action in the poling phase and secondarily for joint stabilisation at pole impact and during the poling phase. A minor role in increasing energy expenditure may be attributed to muscle activation for pole recovery.

Previous studies on muscle activation in NW have produced contradictory results. Our data are in line with those reported by Shim et al. [12] who found similar increases in activation of the *triceps brachii* (+600%) and *latissimus dorsii* muscles (+59%), which are involved in backward arm extension. Two other studies [4,5] reported a 3-fold higher increase in *triceps brachii* muscle activation during NW as compared to conventional walking. Discordant results have been reported for *biceps brachii* muscle activation: Shim et al. (10) found a very high increase in *biceps brachii* muscle activation (+632%), whereas Schiffer et al. [7] found no difference in *biceps brachii* muscle activation between conventional walking and NW. In their study, Schiffer et al. demonstrated, however, that loading the pole increases activation of the *biceps brachii* muscle, confirming its role in pole recovery during the arm swing phase. The same conclusion on the role of the *biceps brachii* muscle was drawn from the observation that simply carrying poles but not using them causes a similar increase in *biceps brachii* muscle activation as when using them (about +50%). The authors suggested that recovery of the mass of the poles was solely responsible for the higher activation of the *biceps brachii* muscle [11].

Smaller increases in muscle activation were also found for walking with hiking poles (9,16). The increase in muscle activation was roughly 50% for the *biceps brachii*, 70% for the *deltoideus anterior*, 100% for the *latissimus dorsii*, and 150% for the *triceps brachii* muscles during uphill walking with poles [11] and 300% for the *triceps brachii* muscle when using a walking pole while carrying a heavy backpack [26]. The lower response reported in the last-mentioned study may have been due to a less intense use of walking poles and to smaller range of movement.

NW was originally developed as summer training exercise for cross-country skiers because it resembles the locomotion pattern of the diagonal stride technique. To date, no direct comparison of muscle activation during these two types of locomotion has been carried out in this context. Studies investigating upper body muscle activity demonstrated considerable trunk and arm muscle activation in double poling cross-country skiing [13,14], with activation exceeding the level reached during maximal voluntary contraction at high-velocity exercise [13]. Comparing muscle-activation patterns in cross-country skiing and NW is difficult owing to the differences in speed of locomotion and the particular joint movement patterns of double poling. What can be hypothesised is that muscular activity is lower in NW than in cross-country skiing.

The only difference we noted in lower limb muscle activation when the subjects walked with poles was a slight but significant reduction in activation of the *gastrocnemius lateralis* muscle, which is activated during the push-off phase to propel the body forward. This may be because the force exerted through the poles relieves part of the propulsion achieved with the leg. Shim et al. [12] found no differences in lower body muscle activation when walking poles were used. In contrast, another study [5] reported significantly lower activation of the *vastus lateralis* and *gastrocnemius* muscles at all walking speeds investigated and of the *biceps femoris* muscle at a very high walking speed. However, because the participants in the study by Shim et al. were not experienced in NW, their poling action was probably not such that it could have an effect on lower limb action. On the basis of our and previously published results, we can say that proper pole technique during NW can provide forward propulsion and reduce *gastrocnemius* muscle action during push off [27].

Different from NW, the relative contribution of the poles to gain forward propulsion in cross-country skiing can range from 20% of total work up to being nearly the only source of propulsion depending on the technique and the conditions considered [22,28]. The amount of force exerted through the pole we noted for NW is comparable to that reported for low-intensity diagonal stride cross-country skiing, and it is 3 times lower than for double poling [14,29]. The larger difference in poling action between NW and cross-country skiing can be ascribed to the shorter poling time in cross-country skiing, resulting in higher power production [14,29].

### Nordic Walking Adaptation to Uphill Walking

Additional power from muscle action is needed for uphill walking. We found that uphill walking increased activation of all the leg muscles investigated. This adaptation was similar during both conventional walking and NW and comparable to that previously reported for upslope walking [16,17,18]. The activity of the *tibialis anterior* muscle, which primarily serves to lift the foot during leg swing during uphill walking, was increased by about 10% between the level and the uphill walking condition. Our observation that the *tibialis anterior* muscle plays a minor role in sustaining the increased incline is shared by other studies that found small [18] or no [16] changes with grade in ankle flexor activity. A greater adaptation of muscular activity was seen for the *gastrocnemius*, *vastus lateralis*, and *biceps femoris* muscles, with an estimated increase of between 60% and 80% during uphill walking. Based on previous studies investigating the effects of slope on leg muscle activation during conventional walking [16,18], we can hypothesise that the leg extensor muscles in particular are activated to meet the demand required by increased slope. The lack of a significant gait x slope interaction suggests, however, that walking with poles does not increase the work required by the leg muscles.

A marked increase in activation of the *biceps brachii*, *trapezius* and *erector spinae* muscles was noted in the uphill walking condition. The 50% increase in activation of the *biceps brachii* muscle during uphill NW can be explained by the need to lift the pole before ground contact to a higher point than during level NW. No increase in muscle activity in force exertion or in force exerted through the pole was found during uphill NW. This suggests that pole use played no role in exploiting the extra mechanical work required to overcome gravity for uphill locomotion. This observation is corroborated by the fact that the differences in oxygen consumption between NW and conventional walking were smaller during uphill walking than level walking and that the differences between walking with and without the poles at 15% incline became even smaller. Importantly, the increase in metabolism during NW at 0% and 15% incline was roughly the same in absolute values, suggesting that the contribution from the upper body during NW remains roughly the same during level and uphill walking. Moreover, It has been demonstrated that walking with trekking poles does not increase the energy cost and that muscular activity is redistributed from the lower to the upper limbs [11].

Interestingly, we found a gait x slope interaction for the *erector spinae* muscle, with greater increases in activation during conventional walking than NW in the uphill condition. The primary function of the e*rector spinae* muscle is to control trunk flexion during gait [30]. However, the epaxial muscles are thought to serve different and somehow conflicting functions in mammalian terrestrial locomotion [31]. Besides mobilising the trunk, and so contribute to propulsion through the production of mechanical work, they also dynamically stabilize the trunk by counteracting movements that are passively induced by external forces or actively produced by antagonist muscles. In uphill trotting dogs, for example, elevated epaxial muscle activity works to stabilise the pelvis against the increased moment imposed by the hindlimb retractor muscles and assists in the production of lateral bending of the trunk [31].

Similar muscular adaptations to meeting increased work demand are seen in stair climbing in humans. When muscle activation patterns during level walking and stair ambulation were compared, the *erector spinae* muscle was found to help the climbing limb swing over the next step, thus elevating the pelvis on this side [32]. Two different mechanisms may underlie the decreased activity of the *erector spinae* muscle with pole use. First, adaptation to uphill walking involves increasing trunk tilt [33], which increases activation of the *erector spinae* muscle [34]. A study on uphill backpacking [26] demonstrated that peak trunk flexion and extension velocities were reduced when hiking poles were used. The authors reported that pole use had a significant effect on reducing trunk movement in the sagittal plane, with less need to actively control trunk flexion by engaging the *erector spinae* muscle. Second, the activity of the *erector spinae* muscle during uphill walking with poles may be decreased due to the propulsive work done by the upper body, which reduces the work of the hip retractor muscles.

## Conclusion

The use of walking poles engages the upper body muscles and elicits higher oxygen consumption during uphill and level walking. The smaller differences in energy expenditure between uphill conventional walking and NW can be explained by the observation that the additional work for climbing against gravity seems to be carried out by the legs during both conventional walking and NW. When performing NW on inclines, the use of the poles does not contribute to the additional work to climb against gravity; however, we may speculate that, if the subjects had been asked to put more effort into poling action than they spontaneously exerted, the energy expenditure would probably have increased, reaching the percent differences between NW and W seen for level walking. Moreover, pole use during uphill walking decreases contraction of the *erector spinae* muscle. From a clinical perspective, the increased *erector spinae* muscle activity associated with conventional uphill walking could lead to muscle overuse and result in low back problems [35]. Since *erector spinae* muscle contraction is decreased during NW, this form of exercise may be suited for people with chronic low back pain and back disorders. The clinical implications of these observations warrant further study.
